# Establishment of a novel cynomolgus monkey model of hyperuricemia

**DOI:** 10.1002/ame2.70128

**Published:** 2026-01-19

**Authors:** Ji‐Wei Wang, Le Zhang, Can Yang, Guan‐Cong Luo, Rui‐Chang Liu, Yan‐Jun Xu, Sheng Cheng, Wen‐Yu Jiang, Richard Ward, Yang Yang, Cheng Xiang, Shu An, Tian‐Rui Xu

**Affiliations:** ^1^ Department of Pharmaceutical Engineering, Faculty of Life Science and Technology Kunming University of Science and Technology Kunming China; ^2^ Department of Primate Disease Model Research, State Key Laboratory of Primate Biomedical Research Kunming University of Science and Technology Kunming China; ^3^ Kunming University of Science and Technology Kunming China; ^4^ Centre for Translational Pharmacology, Institute of Molecular Cell and Systems Biology, College of Medicine University of Glasgow Glasgow UK

**Keywords:** disease model, hyperuricemia, nonhuman primate, uricase

## Abstract

**Background:**

Chronic hyperuricemia is associated with complications such as gout and uric acid nephropathy, but uric acid also exhibits biological activities (e.g., antioxidant effects, potential neuroprotective properties against neurodegenerative diseases). Nonhuman primates are ideal models for studying neurodegenerative diseases; however, existing nonhuman primate hyperuricemia models cannot sustain long‐term elevated serum uric acid levels, nor recapitulate the impaired uric acid excretion observed in clinical hyperuricemic patients.

**Methods:**

First, we detected uricase expression in cynomolgus monkeys and compared it with that in mice. Then, we established a cynomolgus monkey hyperuricemia model by administering a mixture of potassium oxonate, hydrochlorothiazide, and adenine via fruits and vegetables. We further analyzed the regulatory effects of this model on uric acid metabolism (synthesis, degradation, and excretion) and the expression of uric acid transporter genes in the intestine and kidney.

**Results:**

Cynomolgus monkeys express functional uricase, but at a lower level than mice. The established model maintained stable, long‐term hyperuricemia by three mechanisms: increasing intestinal and renal uric acid excretion load, inhibiting hepatic uric acid degradation, and promoting uric acid synthesis. Additionally, the model downregulated the expression of intestinal/renal uric acid‐secreting transporter genes, while upregulating uric acid‐reabsorbing transporter genes.

**Conclusions:**

This novel cynomolgus monkey hyperuricemia model provides a new tool for investigating the association between hyperuricemia and neurodegenerative diseases, and will help clarify the mechanism by which serum uric acid influences cognitive function.

## Graphical abstract

We established a novel hyperuricemia model in cynomolgus monkeys (A) via oral administration of potassium oxonate, hydrochlorothiazide, and adenine mixed in fruits and vegetables. This model (B) targets uric acid synthesis and enzymatic degradation, and (C) regulates renal and intestinal excretion, thereby stably elevating serum uric acid and serving as a reliable tool for hyperuricemia and related neuroprotective research.

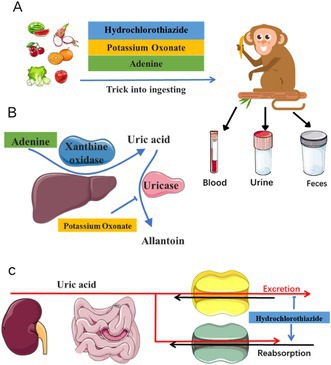

## 1 INTRODUCTION

Uric acid is produced in the liver and then excreted via the intestine and kidneys.^1^, ^2^ Consuming alcohol and fructose leads to a temporary and significant increase in serum uric acid levels.^3^, ^4^ When the serum uric acid level exceeds the normal physiological solubility (6.8 mg/dL), urate crystals form in the joint synovial cavity, causing gout. Although hyperuricemia can lead to a variety of complications, especially gout, uric acid itself plays important physiological regulatory roles, such as regulating vascular osmotic pressure, acting as an antioxidant,^5^ raising blood pressure in higher animals,^6^, ^7^ promoting upright walking, facilitating fat synthesis, and removing oxidative free radicals.^8^ Increasing serum uric acid levels has profound implications for the evolution of higher animal species.

In addition, according to the epidemiological report on hyperuricemia and neurodegenerative diseases, hyperuricemia is negatively correlated with the incidence of these diseases.^9^ For example, high uric acid levels may reduce the incidence of Parkinson's disease and slow its progression, whereas moderately elevated levels of serum uric acid within the normal range could help delay the progression of mild cognitive impairment. However, some studies suggest that the relationship between hyperuricemia and neurodegenerative diseases is not merely a negative correlation but a more complex association. Many studies have explored the correlation between hyperuricemia and neurodegenerative diseases, but the direct relationship between serum uric acid and the central nervous system remains unclear. This represents a gap in the research linking hyperuricemia to neurodegenerative diseases. Developing a suitable animal model of hyperuricemia is crucial for exploring its impact on the onset and progression of neurodegenerative diseases.

Traditional animal models of hyperuricemia primarily use rodents, with modeling methods focused on promoting uric acid synthesis and inhibiting uricase. However, these models fail to mimic the pathological feature of insufficient uric acid excretion, which is seen in over 90% of clinical hyperuricemia patients. Furthermore, there are significant differences in the uric acid metabolism process between rodents and primates. Compared to rodents, primates have an inactivated hepatic uricase, which prevents the oxidation and breakdown of uric acid.^10^ Additionally, rodents are not an ideal model for studying neurodegenerative diseases. Although the neural system structure and function of mice share similarities with those of humans, notable differences still exist. The brain structure and neuronal connectivity of mice do not fully align with those of humans, which may affect the accuracy of disease modeling. Most rodent models used to simulate neurodegenerative diseases often fail to fully replicate the typical pathological features observed in patients. For instance, in Parkinson's disease models, whereas dopaminergic neuron loss is observed, the formation of Lewy bodies (LB), a hallmark pathological feature of the disease, is typically absent. Although well‐established behavioral tests are available for rodent models, they may not fully capture the behavioral and cognitive impairments observed in human patients. Memory and learning tests in mice may not fully replicate the cognitive decline observed in human patients.

In contrast, nonhuman primate models offer clear advantages in exploring the pathogenesis and pathological states of neurodegenerative diseases.^11^ Their brain development, anatomical structures, cognition, and behavioral complexity more closely resemble those of humans. The genetic similarity between nonhuman primates and humans is relatively high, and their pathological development more closely mirrors human diseases, allowing for a more accurate simulation of the pathophysiological processes of human neurodegenerative diseases. Nonhuman primate models are better able to replicate the typical pathological features of neurodegenerative diseases seen in patients. For example, in Parkinson's disease modeling, nonhuman primates may exhibit neuronal loss and the formation of LBs, similar to those observed in humans. Nonhuman primates possess complex cognitive and social behaviors and can perform memory tasks comparable to those of humans. This gives them an advantage in assessing the impact of neurodegenerative diseases on behavior and cognition. Due to their advanced central nervous system, nonhuman primate models can provide more objective evaluations of core behavioral indicators related to brain diseases, such as emotion, social cognition, memory abilities, and fine motor skills. Besides, to establish a clinically relevant hyperuricemia model, we selected cynomolgus monkeys (*Macaca fascicularis*) as experimental animals due to their high genetic homology with humans.^12^

On the contrary, constructing disease models in nonhuman primates is relatively challenging, and the currently reported models of hyperuricemia in nonhuman primates also have disadvantages. The modeling method involves the intraperitoneal injection of adenine, a precursor to uric acid synthesis, into rhesus monkeys.^13^ Using this method, the serum uric acid levels in the induced hyperuricemia model of nonhuman primates increase significantly within 1 h after injection but then return to baseline levels after 4 h. Moreover, the expression of intestinal and renal uric acid transporters in this induced hyperuricemia model of cynomolgus monkeys does not change significantly, which differs from the pathological characteristics of patients with hyperuricemia caused by impaired uric acid excretion.

In our present research, we found the presence of uricase in the liver of cynomolgus monkeys. Therefore, to establish a hyperuricemia cynomolgus monkey model, it is necessary to inhibit uricase activity. Our hyperuricemia cynomolgus monkey model was successfully established by supplementing uric acid precursors, promoting uric acid reabsorption in the intestine and kidney, and inhibiting uric acid enzymatic activity. And the expression of intestinal and renal uric acid–secreting transporter genes is downregulated, whereas that of uric acid–reabsorbing transporter genes is upregulated. Compared to conventional hyperuricemia models, the present model is more efficient, faster, and better able to replicate the pathological characteristics of clinical hyperuricemia. It is an effective tool for exploring the pathological relationship between hyperuricemia and neurodegenerative diseases.

## 2 MATERIALS AND METHODS

### 2.1 Animal studies

Four male cynomolgus monkeys, aged 12 years and weighing >7 kg, were purchased from Yunnan Yingmao Biotechnology Co., Ltd. (DianFaXunFan [2016–01]). These monkeys were healthy, with no severe physical defects or major communicable diseases. The growth and breeding feed for the cynomolgus monkeys, as well as custom‐made high‐purine feed, were both procured from Yunnan Yaling Technology Co., Ltd.

After having arrived at the Kunming University of Science and Technology Experimental Animal Center (SYXK [Dian] K2018‐0008), the cynomolgus monkeys were quarantined and acclimatized for 1 week in the quarantine room before being transferred to the general‐grade animal observation room for future use. The experimental animals were housed in an environment with a temperature range of 64–84°F (18–29°C), a relative humidity of 30%–70%, and a 12‐h light–dark cycle, where they had ad libitum access to food and water.

The cynomolgus monkeys were housed individually and fed twice daily with growth and breeding feed (3% of body weight), once with fruits and vegetables (2%–3% of body weight), and once with commercial snacks. Prior to blood collection, the monkeys were fasted for 12 h. Under the guidance of a veterinarian, the monkeys were restrained using a cage clamp, and 200 μL of Zoletil 50 (Virbac, France) diluent was administered intramuscularly. After a waiting period of 5 min, the monkeys were anesthetized. The hind legs were shaved and disinfected with iodophor, and 2 mL of blood was collected from a suitable vein. The blood samples were allowed to remain at room temperature. Pressure was applied to stop bleeding, and the monkeys were kept in a quiet, warm environment to allow for natural recovery. After recovery, water and fruits were provided to replenish their intake.

### 2.2 Induction of hyperuricemia model in cynomolgus monkeys

Potassium oxonate (S17112, Shanghai Yuanye Bio‐Technology Co., Ltd) and composite feed containing uric acid (U2625‐25G, Sigma), adenine (YB24793, Hunan Yunbang Bio), and yeast powder (MLP0021B, Shanghai Yuanye Bio‐Technology Co., Ltd) were used in the induction phase. The model was established for 1 month by adding potassium oxonate (0.5 mg/kg of body weight), uric acid (2.5 mg/kg), adenine (0.6 mg/kg), and yeast powder (7 mg/kg) to the feed, in combination with 10% fructose water.

To avoid potential interactions between different modeling methods, a new modeling induction can be performed only after a 2‐week break from the previous modeling. This was followed by the daily water intake limit induction phase. Based on the individual daily water intake variations prior to modeling, an upper limit of 125 mL/day was set for the daily water intake of each cynomolgus monkey, while maintaining the same daily amounts of fruits and vegetables in their diet. The model was followed for a period of 1 month under these conditions.

The combined setting of potassium oxonate feed and the upper limit of daily water intake served as the induction phase. The adenine and uric acid that could affect the feeding behavior of cynomolgus monkeys were removed from the feed, and in combination with setting an upper limit for daily water intake, the modeling was conducted for a period of 1 month.

The combination of potassium oxonate, hydrochlorothiazide (H2910, Sigma), and an upper limit for daily water intake served as the induction phase. The feed was supplemented with potassium oxonate (0.5 mg/kg) and hydrochlorothiazide (0.4 mg/kg), and in combination with an upper limit set for daily water intake, the modeling was conducted for a period of 1 month.

Fruit‐ and vegetable‐based bait used to administer potassium oxonate and hydrochlorothiazide, in combination with an upper limit for daily water intake, served as the induction phase. For a period of 1 month, potassium oxonate (0.5 mg/kg) and hydrochlorothiazide (0.4 mg/kg) were mixed in fruits and vegetables (2%–3%) and combined with an upper limit set for daily water intake to establish the model.

The increase in doses of potassium oxonate and hydrochlorothiazide, along with the removal of the upper limit for daily water intake, served as the modified induction phase. In this modified induction phase, the doses of potassium oxonate and hydrochlorothiazide were doubled compared to the previous protocol, while maintaining all other operational steps as described previously.

The combination of potassium oxonate, hydrochlorothiazide, and adenine served as the induction phase. Adenine (0.2 mg/kg) was added to the feed, with other operational steps consistent with those described earlier.

### 2.3 Statistics

All quantitative data were presented as mean ± standard deviation (SD), and the data were analyzed using GraphPad Prism, version 6 (GraphPad Software, San Diego, CA, USA). Statistically significant differences in uric acid levels in serum, urine, and feces; daily urine and feces output; 24‐h intestinal and renal uric acid excretion; and expression levels of hepatic urate oxidase (UO) and xanthine oxidase (XO) were assessed using an unpaired two‐tailed *t*‐test. The expression levels of intestinal and renal uric acid transporters were assessed using two‐way analysis of variance (ANOVA). Statistical differences are indicated as follows: *p < 0.05, **p < 0.01, ***p < 0.005, and ****p < 0.001.

## 3 RESULTS

### 3.1 The expression levels of uricase and XO in the liver of cynomolgus monkeys were lower than those in mice

**FIGURE 1:**
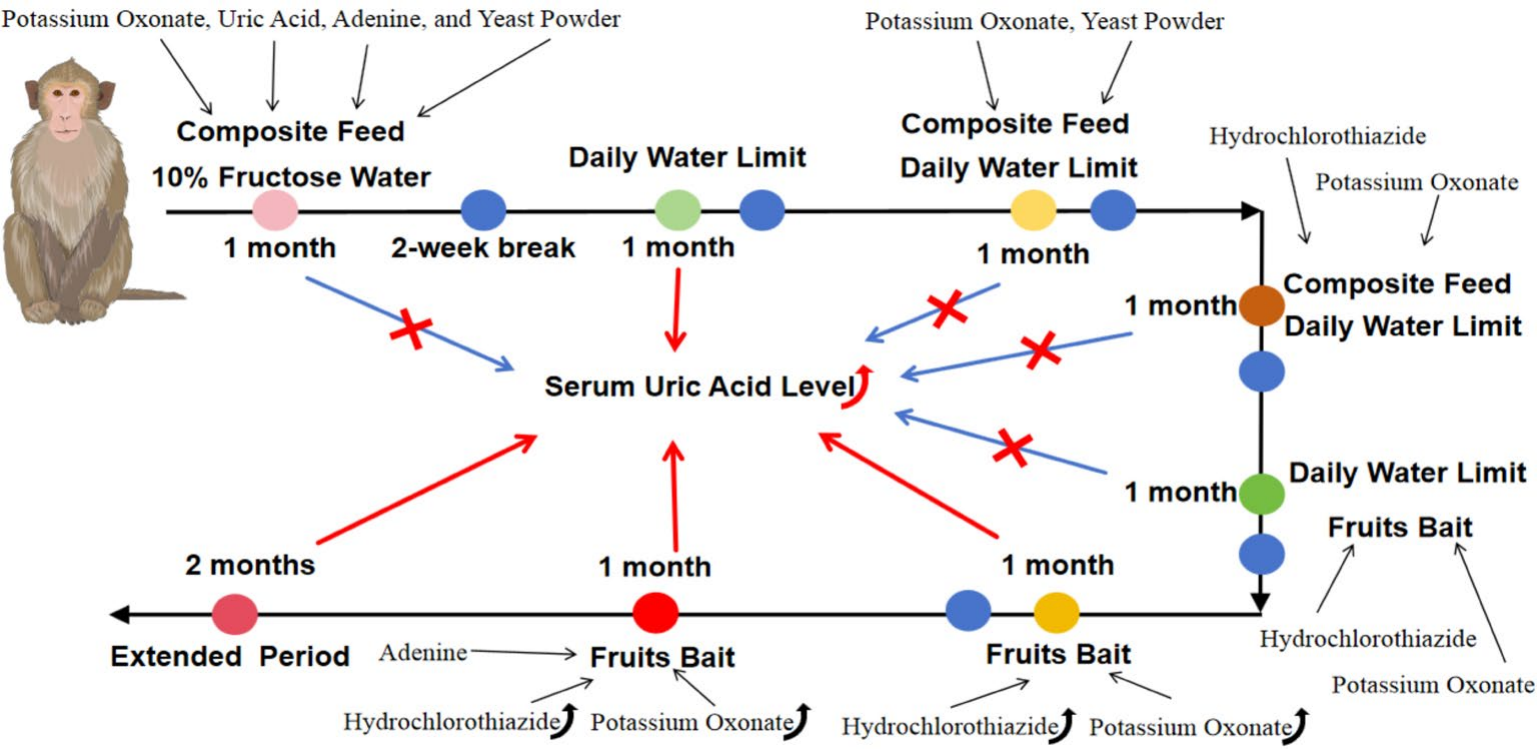
Induction technique road map for hyperuricemia model in cynomolgus monkeys. During the eight‐stage optimization process of hyperuricemia cynomolgus monkey model establishment, the stage indicated by the blue arrow did not result in an elevation of serum uric acid levels in cynomolgus monkeys. However, the stages indicated by the red arrows can all effectively increase the serum uric acid levels in cynomolgus monkeys.

**FIGURE 2:**
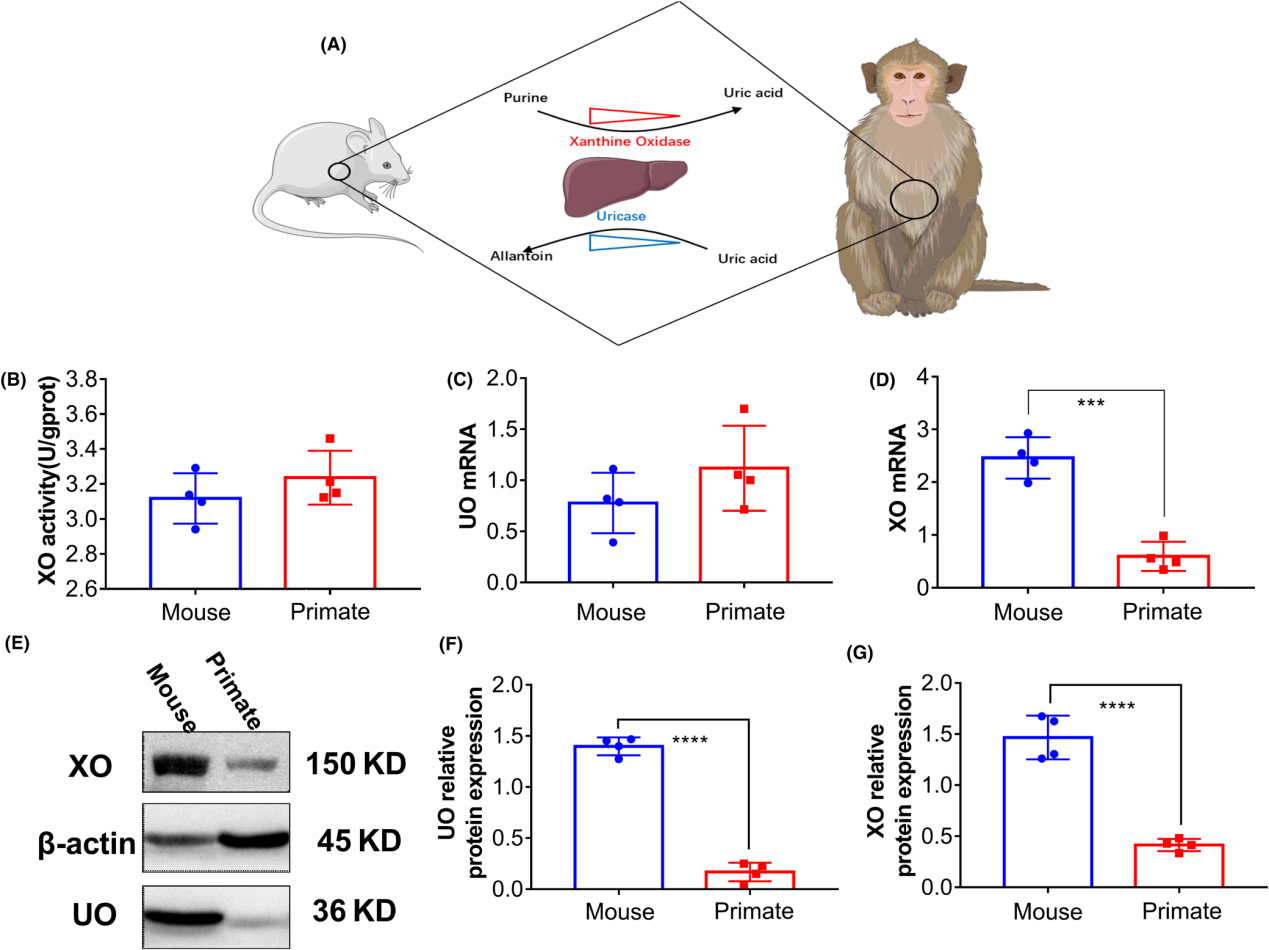
The expression levels of uricase and xanthine oxidase (XO) in the liver of cynomolgus monkeys are lower than those in mice. (A) Schematic diagram. (B) The activity of XO in the liver of cynomolgus monkeys is comparable to that of mice (*n* = 4). (C) The transcriptional level of urate oxidase (UO) in the liver of cynomolgus monkeys is higher than that of mice, but the difference is not statistically significant (*n* = 4). (D) The transcriptional level of XO in the liver of cynomolgus monkeys is significantly lower than that of mice. Results are represented as mean ± SD (standard deviation); differences between groups were analyzed using the *t*‐test (***p < 0.005, *n* = 4). (E) The significantly lower expression levels of UO and XO in the liver of cynomolgus monkeys compared to mice (*n* = 4). (F, G) Quantitative analysis of UO and XO expression levels in the liver of cynomolgus monkeys. Results are represented as mean ± SD; differences between groups were analyzed using the *t*‐test (****p < 0.001, *n* = 4).

The schematic diagram of animal model establishment is shown in Figure 1. As illustrated in Figure 2A mice and cynomolgus monkeys express uricase (UO) and xanthine oxidase (XO) in the liver, but levels of UO and XO in cynomolgus monkeys are markedly lower than that in mice. A liver biopsy performed on cynomolgus monkeys showed that uricase is expressed in the liver. Compared with rodents, there was no significant difference in XO activity in the liver of cynomolgus monkeys (Figure 2B). The transcription (Figure 2C,D) and expression (Figure E–G) levels of XO in the liver of cynomolgus monkeys were significantly lower than those in mice. This suggested that, compared with mice, cynomolgus monkeys have a lower level of uric acid synthesis. And uric acid is metabolized by uricase, which is why it is difficult to establish hyperuricemia in cynomolgus monkeys.

### 3.2 Restricting the upper limit of daily water intake of cynomolgus monkeys can increase the level of serum uric acid

Based on the observation that cynomolgus monkeys retain uricase, we attempted to develop the hyperuricemia model by supplementing their feed with potassium oxonate, uric acid, adenine, and yeast extract, along with 10% fructose drinking water for 1 month. We found that there was no significant change in serum uric acid levels in the monkeys compared to pre‐modeling levels (Figure S1). However, due to the introduction of fructose water during the modeling process, the daily water intake and urine output of the monkeys increased significantly, with urine uric acid level and 24‐h renal uric acid excretion increasing significantly. At this stage, the excessive consumption of fructose water by the cynomolgus monkeys led to increased renal uric acid excretion, which failed to elevate serum uric acid levels. This observation suggested that restricting daily water intake may contribute to the elevation of serum uric acid levels.

**FIGURE 3:**
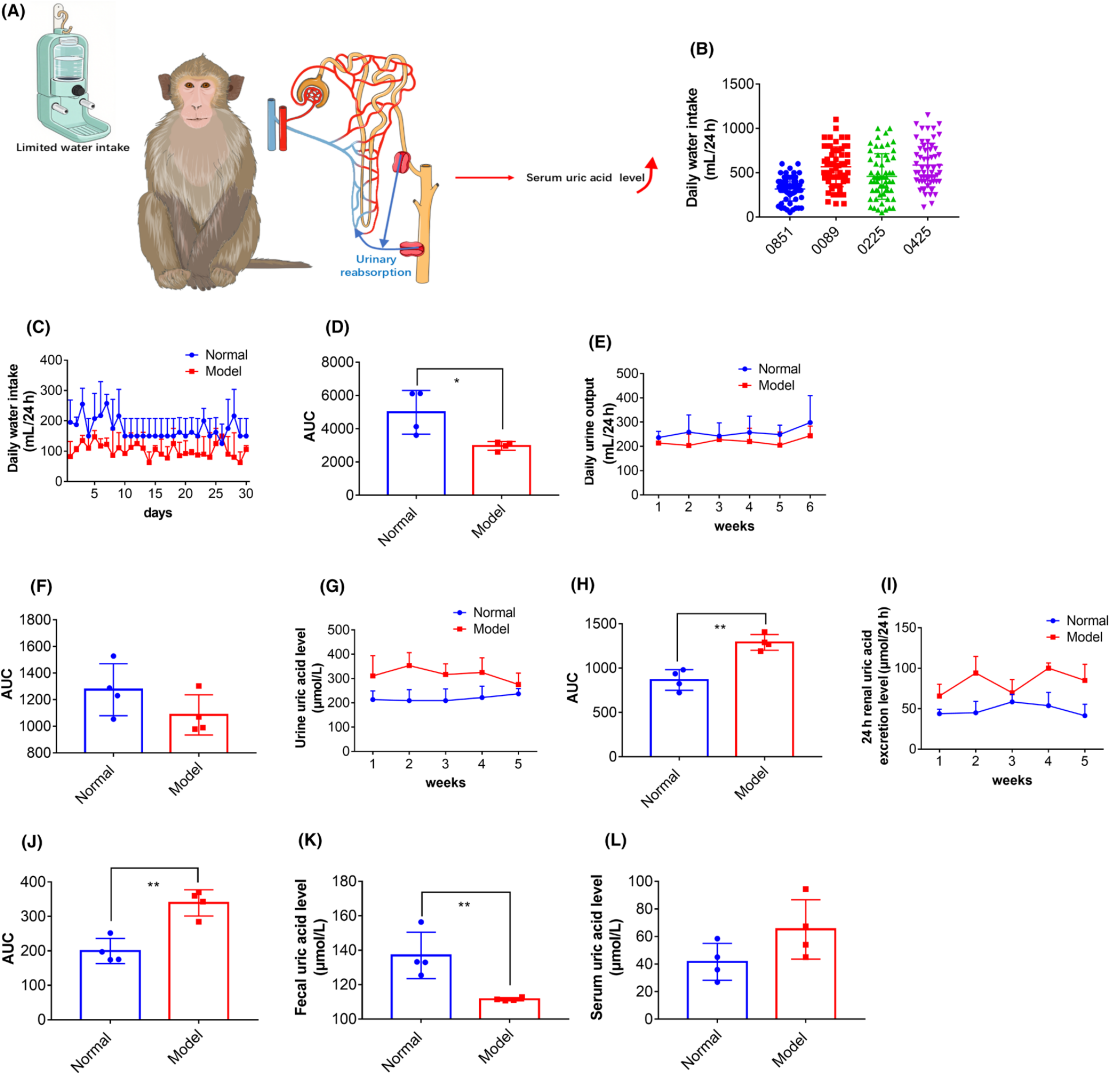
Restricting the upper limit of the daily water intake of cynomolgus monkeys can increase the level of serum uric acid. (A) Schematic diagram. (B) The daily water intake of each cynomolgus monkey during the adaptive feeding stage. (C, D) The daily water intake of cynomolgus monkeys decreased. Results are represented as mean ± SD (standard deviation); differences between groups were analyzed using the *t*‐test (*p < 0.05, *n* = 4). (E, F) The daily urine output of cynomolgus monkeys decreased after an upper limit was set for their daily water intake. (G, H) Significant increase in urine uric acid level of cynomolgus monkeys after setting an upper limit for daily water intake. Results are represented as mean ± SD; differences between groups were analyzed using the *t*‐test (**p < 0.01, *n* = 4). (I, J) Significant increase in 24‐h uric acid excretion in the kidneys of cynomolgus monkeys after setting an upper limit for daily water intake. Results are represented as mean ± SD; differences between groups were analyzed using the *t*‐test (**p < 0.01, *n* = 4). (K) Significant decrease in uric acid level in the feces of cynomolgus monkeys after setting an upper limit for daily water intake. Results are represented as mean ± SD; differences between groups were analyzed using the *t*‐test (**p < 0.01, *n* = 4). (L) The serum uric acid level of cynomolgus monkeys increased, but not significantly, after an upper limit was set for daily water intake.

As illustrated in Figure 3A, which depicts water intake restriction and uric acid metabolism in cynomolgus monkeys, we established individual water intake upper limits based on excessive sugared water consumption, while accounting for daily intake variability (Figure 3B). To eliminate the influence of allopurinol, uric acid, adenine, and yeast extract in the feed, we conducted a 1‐month modeling period. During this period, we monitored the uric acid levels in the serum, urine, and feces of the cynomolgus monkeys to evaluate the modeling effects. After 1 month, we observed that the daily urine output of the cynomolgus monkeys decreased (Figure 3C–F), and the urine uric acid level increased significantly (Figure 3G,H). The 24‐h renal uric acid excretion increased significantly (Figure 3I,J), and the uric acid level in feces decreased (Figure 3K). The serum uric acid level increased but not significantly (Figure 3L). Reducing the daily water intake of cynomolgus monkeys facilitated the reabsorption of uric acid in the kidneys. However, the lack of precursors for uric acid synthesis and the absence of uricase inhibitors in the diet did not significantly elevate the serum uric acid level in the cynomolgus monkeys.

### 3.3 Inhibiting uric acid degradation and decreasing excretion through the intestine and kidney are conducive to increasing the level of uric acid in the serum of cynomolgus monkeys

Because uric acid and adenine in the feed affected the feeding behavior of cynomolgus monkeys, we eliminated uric acid and adenine from the feed and set an upper limit on daily water intake for modeling for 1 month. We found that the uric acid excretion level in the kidneys of cynomolgus monkeys was not effectively restricted, resulting in a decrease in serum uric acid level (Figure S2).

To inhibit the excretion of uric acid in the kidneys, we added hydrochlorothiazide to the feed of cynomolgus monkeys, combined with setting an upper limit on daily water intake for modeling for 1 month. We found that the excretion of uric acid in the kidneys of cynomolgus monkeys was restricted by hydrochlorothiazide, but this effect had no impact on the excretion of uric acid in the intestines, resulting in a decrease in serum uric acid level in cynomolgus monkeys (Figure S3).

Furthermore, during the modeling process, we observed that the addition of potassium oxonate and hydrochlorothiazide to the feed severely affected the feeding interest of cynomolgus monkeys. Therefore, we added potassium oxonate and hydrochlorothiazide to the daily intake of fruits and vegetables for cynomolgus monkeys, using the fruits as bait to induce the monkeys to ingest uric acid hydrolysis inhibitor and uric acid excretion inhibitor. We found that the excretion of uric acid in the kidneys of cynomolgus monkeys was restricted, but the remaining uric acid was compensatorily excreted through the intestines, resulting in a decrease in serum uric acid level in cynomolgus monkeys (Figure S4).

**FIGURE 4:**
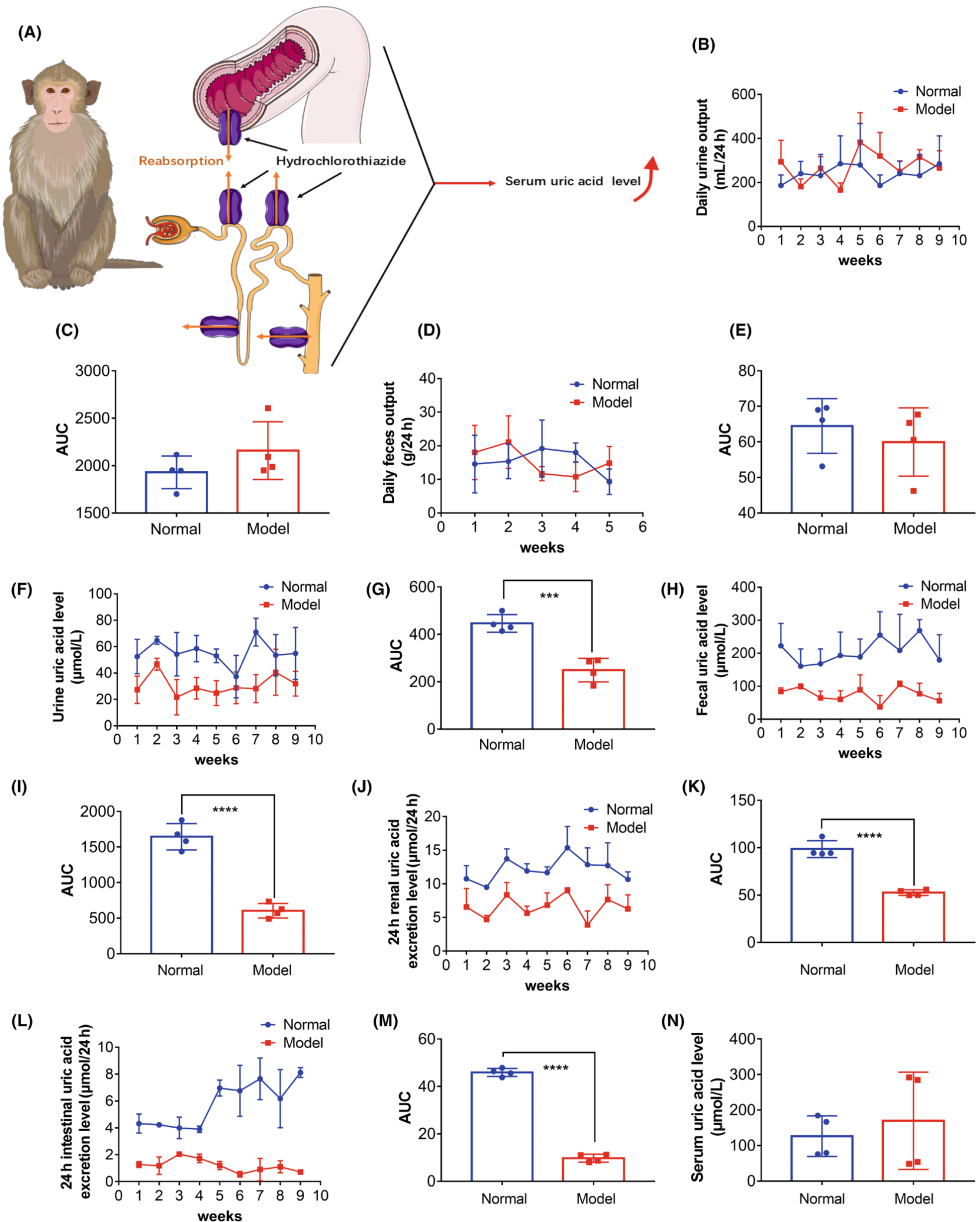
Inhibiting uric acid degradation and decreasing the excretion of the intestine and kidney are conducive to increasing the level of uric acid in the serum of cynomolgus monkeys. (A) Schematic diagram. (B–E) After the upper limit of daily water intake was increased and oxonate potassium and hydrochlorothiazide was administered via fruits and vegetables to cynomolgus monkeys, there were no significant changes in the daily urine output and feces excretion of cynomolgus monkeys. (F–I) The levels of uric acid in the urine and feces of cynomolgus monkeys decreased significantly. Results are represented as mean ± SD (standard deviation); differences between groups were analyzed using the *t*‐test (***p < 0.005, ****p < 0.001, *n* = 4). (J–M) Significant decrease in 24‐h uric acid excretion in the kidneys and intestines of cynomolgus monkeys. Results are represented as mean ± SD; differences between groups were analyzed using the *t*‐test (****p < 0.001, *n* = 4). (N) The serum uric acid level of cynomolgus monkeys increased but not significantly.

Additionally, the combination of increasing diuretic dose and limiting daily water intake may cause irreversible renal damage in cynomolgus monkeys; thus, we removed the water intake limit, as illustrated in Figure 4A. After 1 month of modeling, we found that there were no significant differences in the daily urine (Figure 4B,C) and fecal output (Figure 4D,E) of cynomolgus monkeys, while the uric acid levels in the urine and feces of cynomolgus monkeys decreased (Figure 4F–I), and the 24‐h intestinal and renal uric acid excretion was significantly reduced (Figure 4J–M), but the increase in serum uric acid level was not significant (Figure 4N).

### 3.4 Inhibiting uric acid degradation and excretion, while promoting the synthesis of uric acid, is conducive to increasing the serum uric acid level in cynomolgus monkeys

**FIGURE 5:**
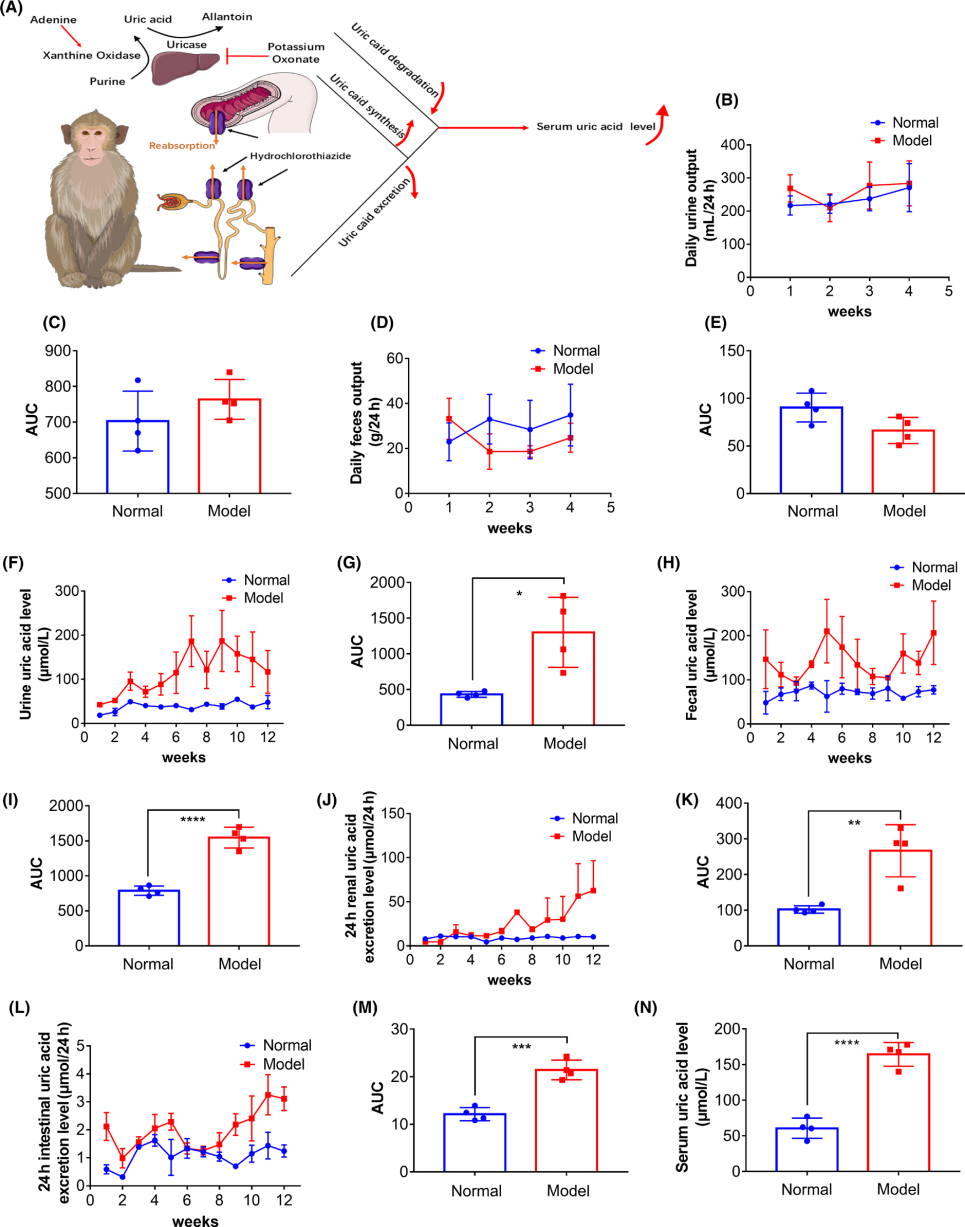
Inhibiting uric acid degradation and intestinal and renal excretion, while promoting the synthesis of uric acid, is conducive to increasing the serum uric acid level in cynomolgus monkeys. (A) Schematic diagram. (B–E) After the cynomolgus monkeys were administered oxonate potassium, hydrochlorothiazide, and adenine via fruits and vegetables, there were no significant changes in the daily urine and feces output. (F–I) The levels of uric acid in the urine and feces of cynomolgus monkeys increased significantly. Results are represented as mean ± SD (standard deviation); differences between groups were analyzed using the *t*‐test (*p < 0.05, ****p < 0.001, *n* = 4). (J–M) Significant increase in 24‐h uric acid excretion of the kidneys and intestines of cynomolgus monkeys. Results are represented as mean ± SD; differences between groups were analyzed using the *t*‐test (**p < 0.01, ***p < 0.005, *n* = 4). (N) The serum uric acid level of cynomolgus monkeys significantly increased. Results are represented as mean ± SD; differences between groups were analyzed using the *t*‐test (****p < 0.001, *n* = 4).

We added adenine to the daily intake of fruits and vegetables of cynomolgus monkeys and maintained the modeling induction with oxonic potassium and hydrochlorothiazide for 1 month (Figure 5A). We found that there were no significant changes in the daily urine output and feces excretion of cynomolgus monkeys (Figure 5B–E), whereas the uric acid levels in urine and feces increased significantly (Figure 5F–I). The 24‐h uric acid excretion through the intestines and kidneys also increased significantly (Figure 5J–M), resulting in a significantly increased intestinal and renal uric acid excretion load and serum uric acid level in cynomolgus monkeys (Figure 5N).

**FIGURE 6:**
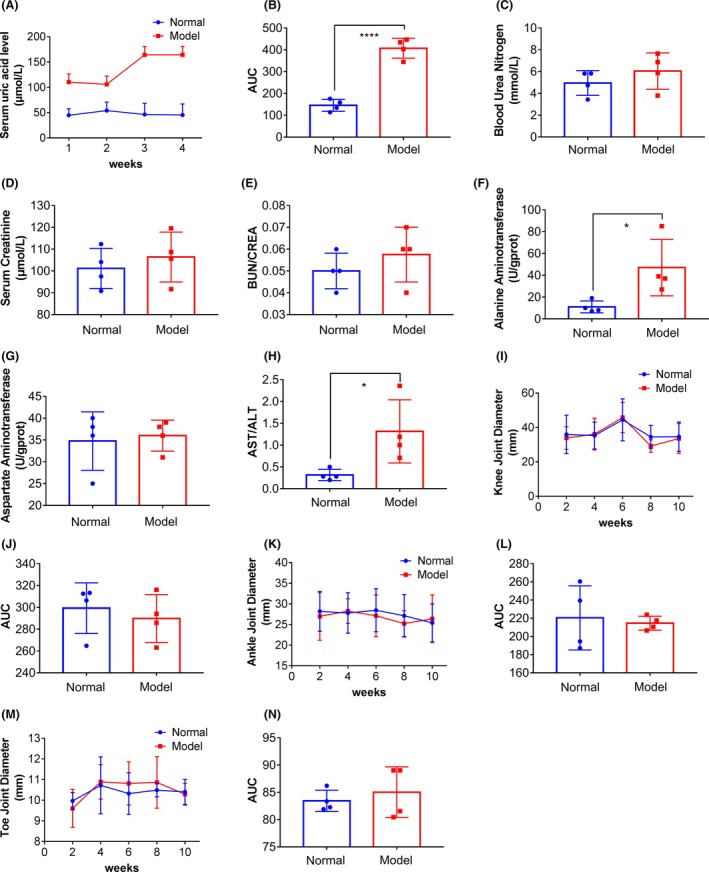
The serum uric acid level of the cynomolgus monkey model with hyperuricemia can be stably maintained for a long period of time. (A, B) After the modeling time was extended for another 1 month, the serum uric acid level in cynomolgus monkeys remained stable. Results are represented as mean ± SD (standard deviation); differences between groups were analyzed using the *t*‐test (****p < 0.001, *n* = 4). (C–E) There were no significant changes in serum urea nitrogen and creatinine levels in cynomolgus monkeys. (F–H) The elevation of ALT levels in the serum of cynomolgus monkeys may be attributed to their long‐term administration of oxonic potassium salt and hydrochlorothiazide, whereas there is no significant change in AST levels. Results are represented as mean ± SD; differences between groups were analyzed using the *t*‐test (*p < 0.05, *n* = 4). (I–N) There were no significant changes in the diameters of the knee, ankle, and toe joints in cynomolgus monkeys.

To investigate the stability of this hyperuricemia cynomolgus monkey model, we extended the modeling period for another 1 month and collected blood from the cynomolgus monkeys. We found that the serum uric acid level of the cynomolgus monkeys increased significantly (Figure 6A,B), indicating that the symptoms of hyperuricemia could be maintained stably for a long time. In addition, we examined the liver and kidney functions of the cynomolgus monkeys and found that long‐term hyperuricemia modeling had no significant effect on their kidney function (Figure 6C–E), but the serum alanine aminotransferase level in the cynomolgus monkeys increased (Figure 6F–H), which may be due to the long‐term administration of inducers such as oxonic potassium, hydrochlorothiazide, and adenine. However, after 1 week cessation of modeling, we found that the aspartate aminotransferase/alanine aminotransferase (AST/ALT) value recovered (Figure S5). Furthermore, we observed symptoms of arthritis in the lower limbs of the cynomolgus monkeys (Video S1–, S4), but there were no significant changes in the diameters of knees, ankles, and toes in the cynomolgus monkeys (Figure 6I–N), and uric acid crystals were not detected in the joint fluids of the cynomolgus monkeys (results not shown).

**FIGURE 7:**
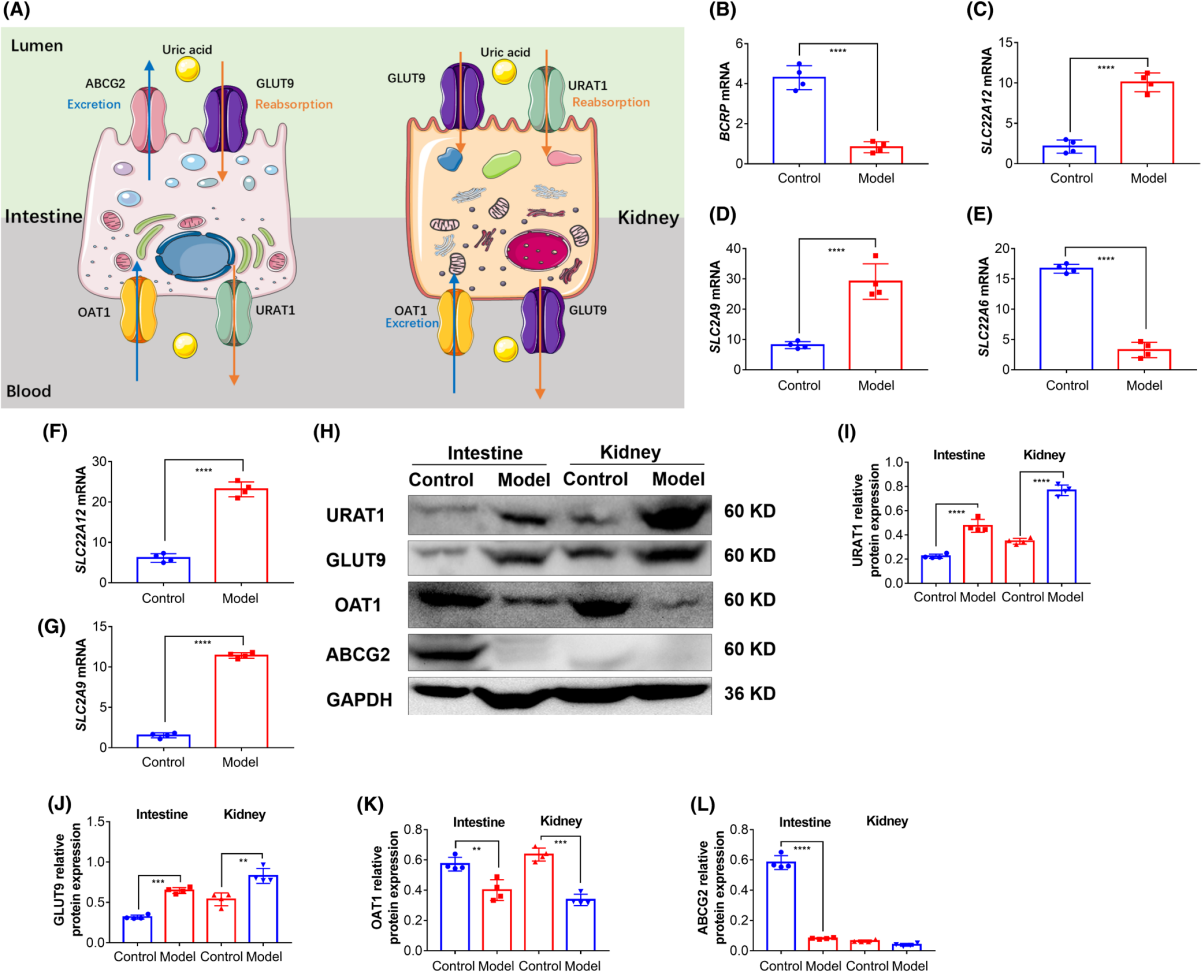
The expression of the intestinal and renal uric acid secretory genes was downregulated, whereas that of the uric acid reabsorption genes was upregulated in hyperuricemic cynomolgus monkeys. (A) Schematic diagram. (B–D) Transcription level of intestinal uric acid transporter genes in cynomolgus monkeys. Results are represented as mean ± SD (standard deviation); differences between groups were analyzed using the *t*‐test (****p < 0.001, *n* = 4). (E–G) Transcription level of renal uric acid transporter genes in cynomolgus monkeys. Results are represented as mean ± SD; differences between groups were analyzed using the *t*‐test (****p < 0.001, *n* = 4). (H) Expression level of intestinal and renal uric acid transporter proteins in cynomolgus monkeys. (I–L) Grayscale scanning of the expression levels of URAT1, GLUT9, OAT1, and ABCG2. Results are represented as mean ± SD; differences between groups were analyzed using two‐way ANOVA (analysis of variance) (**p < 0.05, ***p < 0.005, ****p < 0.001, *n* = 4).

On the contrary, by examining the expression levels of uric acid transporter genes in the intestinal and renal biopsy samples from cynomolgus monkeys before and after model establishment, we observed that after 2 months of inducing cynomolgus monkeys to ingest oxonic acid potassium, hydrochlorothiazide, and adenine via fruits and vegetables, the transcription levels of intestinal and renal uric acid secretory genes (*BCRP*, *SLC22A6*) significantly decreased (Figure 7A), whereas the transcription levels of uric acid reabsorption transporter genes (*SLC22A12*, *SLC2A9*) significantly increased (Figure 7B–G). Additionally, the expression levels of the intestinal and renal uric acid secretory proteins (ABCG2, OAT1) were reduced, and the expression levels of the uric acid reabsorption proteins (URAT1, GLUT9) were upregulated (Figure 7H–L).

## 4 DISCUSSION

Uric acid plays a crucial role in various physiological processes, including antioxidation, regulation of blood osmotic pressure, and promotion of fat synthesis. Elevating serum uric acid levels has contributed to the evolutionary selection of higher animals. Maintaining a high level of serum uric acid can provide several advantages, such as supplying reducing power during periods of food scarcity, helping to maintain body temperature during severe winters, regulating blood osmotic pressure to elevate blood pressure, and facilitating the upright walking of primates.^14^ In recent years, the number of patients suffering from hyperuricemia has surged, particularly those with gouty arthritis and gouty nephropathy, which are common complications of hyperuricemia.^15^ Research on the pathogenesis and treatment options of hyperuricemia and gout has also been increasing. Furthermore, there have been increasing reports suggesting a negative correlation between hyperuricemia and the incidence of neurodegenerative diseases.^16^, ^17^ In vitro experiments have also demonstrated the antioxidant activity of uric acid, showing that the clearance of oxidative free radicals in neuronal cells is influenced by uric acid.^18^ However, there are a relatively few in vivo studies on the effects of hyperuricemia on the occurrence and development of neurodegenerative diseases, and research explaining the direct relationship between them remains superficial.

It has been reported that nonhuman primate models of hyperuricemia can currently be established, primarily through intraperitoneal injection of adenine. Using this method, the serum uric acid level in the hyperuricemic rhesus monkey model increases gradually at 1 h of injection and then returns to baseline levels after 4 h.^13^ No differences in the expression of intestinal and renal urate transporters were observed in the rhesus monkeys, and no data were provided on the level of intestinal and renal uric acid excretion. The serum uric acid level in the hyperuricemic rhesus monkey model obtained using this method cannot be maintained over the long term, and there is no detailed information on intestinal and renal uric acid excretion. Therefore, it is not possible to replicate the pathological characteristics of uric acid excretion disorder observed in clinical hyperuricemia patients.

**FIGURE 8:**
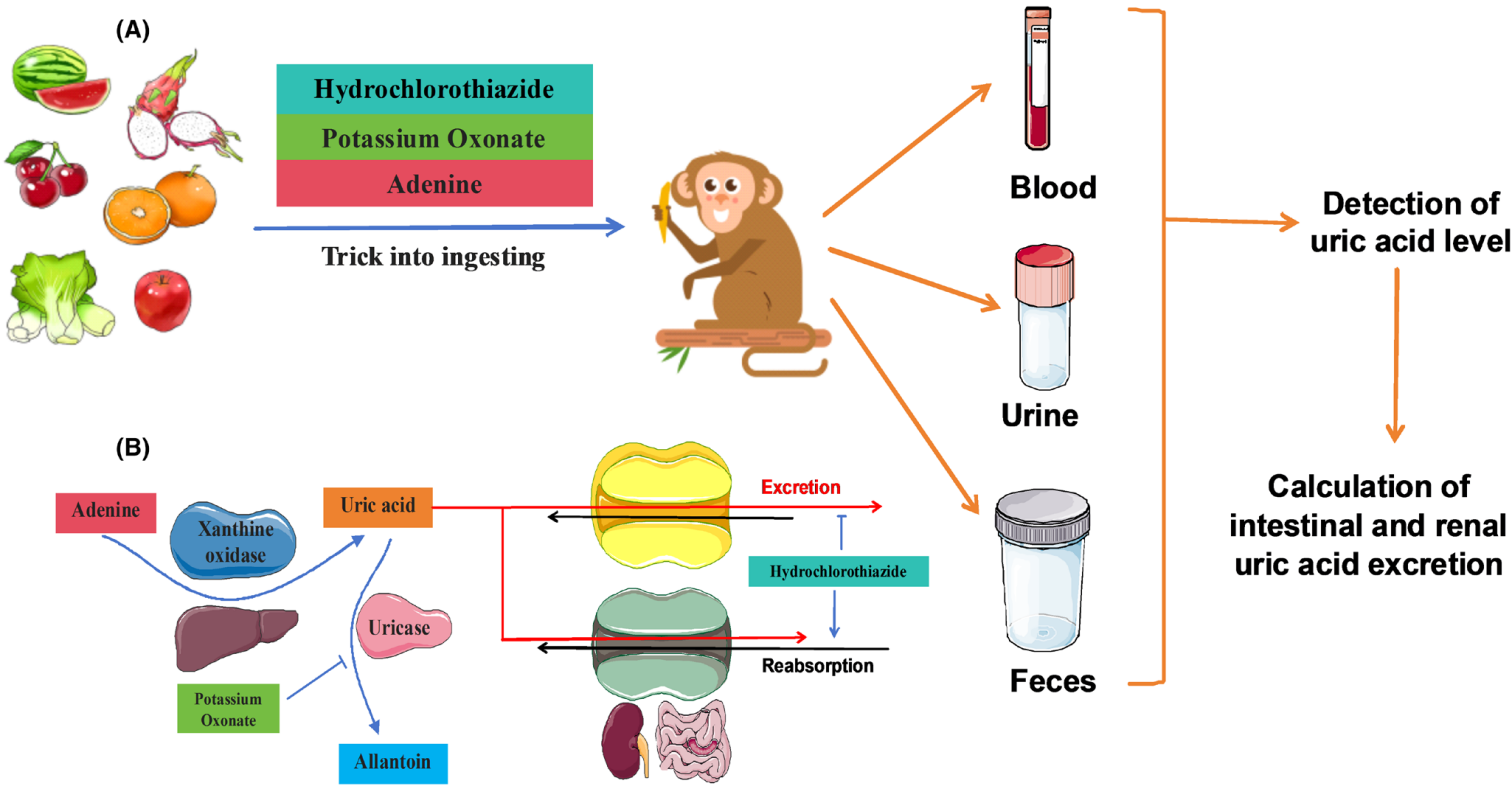
Schematic diagram of a cynomolgus monkey model of hyperuricemia with increased intestinal and renal uric acid excretion load. (A) How to induce hyperuricemia in a cynomolgus monkey model. Oxonic acid, hydrochlorothiazide, and adenine were mixed in seasonal fruits and vegetables to trick the cynomolgus monkeys into ingesting them. During the 1‐month modeling period, blood, urine, and feces were collected from the cynomolgus monkeys, and the uric acid level was measured to calculate the 24‐h intestinal and renal uric acid excretion levels of the cynomolgus monkeys. (B) Why does the hyperuricemia model work. Purines are sequentially converted into uric acid and allantoin under the action of xanthine oxidase and uricase in the liver, and uric acid is excreted through intestinal and renal uric acid transporters. Oxonic acid potassium can inhibit uricase activity; hydrochlorothiazide inhibits intestinal and renal uric acid excretion and promotes uric acid reabsorption; and the combined model is conducive to maintaining serum uric acid levels in hyperuricemic cynomolgus monkeys.

In this study, we found that cynomolgus monkeys retain uricase in their livers, which decomposes uric acid, making the use of the uricase inhibitor oxonic acid necessary. The adenine and uric acid itself significantly affected the feeding behavior of cynomolgus monkeys. Therefore, we used fruits and vegetables to encourage the ingestion of the modeling agents, tailored to the preferences of each monkey. We attempted to limit renal uric acid excretion in cynomolgus monkeys by restricting their daily water intake. However, the effect was minimal, and the intestinal uric acid excretion pathway in cynomolgus monkeys was compensatorily activated, leading to failure in inducing the hyperuricemia model. Therefore, by using the diuretic hydrochlorothiazide to limit intestinal and renal uric acid excretion in cynomolgus monkeys, combined with uricase inhibition and adenine supplementation (Figure 8), we successfully established a hyperuricemic cynomolgus monkey model. By upregulating the expression of the intestinal and renal uric acid reabsorption genes and downregulating the expression levels of the uric acid secretory genes, the ability of the intestine and kidney to excrete uric acid was inhibited, thereby maintaining stable serum uric acid levels over the long term. On the contrary, we found that long‐term modeling led to serum ALT increase in cynomolgus monkeys, but such change was reversible, with the increase starting to recover 1 week after cessation of modeling. Therefore, if stable serum uric acid levels in cynomolgus monkeys are maintained, intermittent modeling is beneficial for protecting their livers and preventing anorexia.

There are several limitations to this study. First, the method of inducing cynomolgus monkeys to ingest modeling agents using fruits and vegetables involves frequent adjustments. Certain fruits and vegetables play a crucial role in regulating the production of uric acid and inhibiting its excretion.^19^, ^20^ Fruits high in fructose can promote uric acid synthesis in cynomolgus monkeys,^21^ whereas fruits rich in reducing acids inhibit uric acid accumulation.^22^ Second, modeling agents can also be administered to cynomolgus monkeys through commercial snacks, such as dried fish and shrimp, and bread, which provide the necessary precursors for uric acid synthesis. However, no quantitative and classification studies have been conducted on uric acid synthesis mediated by fruits, vegetables, and snacks. Third, although a hyperuricemia model has been successfully established in cynomolgus monkeys, exploring its impact on cognitive behavior remains at a bottleneck stage. Although uric acid in the circulatory system cannot directly cross the blood–brain barrier to enter the central nervous system, some transporters are capable of transporting uric acid along with other organic acids.^23^ It is important to continue optimizing the model to enhance the ability of circulatory uric acid to cross the blood–brain barrier and investigate its long‐term (6‐month) effects on the central nervous system; besides tracking the dynamic changes in serum uric acid, we will systematically collect neurodegeneration‐related pathological indicators through multidimensional studies, including conducting a battery of behavioral and cognitive assessments to evaluate whether chronic hyperuricemia induces progressive cognitive decline^24^ or neurobehavioral abnormalities, performing neuropathological examinations via immunohistochemistry and confocal microscopy to investigate pathological changes in key brain regions,^25^ and measuring levels of oxidative stress markers, neuroinflammatory cytokines, and synaptic proteins in brain tissues^26^ to explore whether uric acid–mediated pathways contribute to neurodegenerative processes, which will collectively determine the relationship between elevated brain uric acid levels and neurodegenerative changes and provide comprehensive evidence for the model's utility in investigating uric acid–related neurological disorders.

In conclusion, we successfully established a hyperuricemia model in cynomolgus monkeys using a combination of fructose water, oxonic acid potassium, hydrochlorothiazide, and adenine. This model differs from the traditional hyperuricemia cynomolgus monkey model, which is induced by an intraperitoneal injection of adenine. It features stable and long‐term serum uric acid levels, and has minimal impact on the liver and kidney functions of cynomolgus monkeys. Additionally, the cynomolgus monkeys were selected based on two pivotal considerations: (1) their uricase expression level is low compared with mice as our results show, and (2) primate‐specific neurovascular coupling allows accurate assessment of uric acid's dual roles in neuroprotection and potential neurotoxicity.^27^ Besides, the present research provides an ideal primate platform for investigating the intricate balance between uric acid's neuroprotective antioxidant properties and its potential neurotoxic inflammatory effects in the central nervous system, thereby bridging critical gaps in translational research between preclinical studies and human clinical applications.

## AUTHOR CONTRIBUTIONS

**Tian‐Rui Xu:** Funding acquisition. **Ji‐Wei Wang:** Writing – original draft. **Le Zhang:** Methodology. **Can Yang:** Formal analysis. **Guan‐Cong Luo:** Investigation. **Rui‐Chang Liu:** Data curation. **Yan‐Jun Xu:** Resources. **Sheng Cheng:** Software. **Wen‐Yu Jiang:** Validation. **Richard Ward:** Writing – review and editing. **Yang Yang:** Visualization. **Cheng Xiang:** Supervision. **Shu An:** Project administration.

## FUNDING INFORMATION

This study was supported by the National Natural Science Foundation of China (grant nos 32160157 and 82160564), the Major Science and Technology Project of Yunnan Province (grant no. 202202AG050008), and the Yunnan “Xingdian Talent Program” Yunling Scholar Project (grant no. CA24129L025A).

## CONFLICT OF INTEREST STATEMENT

The authors declare that they have no competing interests.

## ETHICS STATEMENT

Ethics approval for this study was obtained from the Animal Ethics Review Committee of the Experimental Animal Management Center, Kunming University of Science and Technology (Approval Number: SLWH (Dian) K2020‐0001). All animal experiments were conducted in strict accordance with the ethical guidelines and regulations for the care and use of laboratory animals.

## Supporting information


Figure S1.

Figure S2.

Figure S3.

Figure S4.

Figure S5.

**Table S1.** Sequences of primer used in qPCR.

Video S1.

Video S2.

Video S3.

Video S4.

## References

[1] Taniguchi A , Kamatani N . Control of renal uric acid excretion and gout. Curr Opin Rheumatol. 2008;20(2):192‐197. 18349750 10.1097/BOR.0b013e3282f33f87

[2] Beck LH . Clinical disorders of uric acid metabolism. Med Clin North Am. 1981;65(2):401‐411. 6894473 10.1016/s0025-7125(16)31531-0

[3] Zhang Y , Woods R , Chaisson CE , et al. Alcohol consumption as a trigger of recurrent gout attacks. Am J Med. 2006;119(9):800.e13‐2. 10.1016/j.amjmed.2006.01.020 16945617

[4] Sharpe CR . A case‐control study of alcohol consumption and drinking behaviour in patients with acute gout. Can Med Assoc J. 1984;131(6):563‐567. 6478339 PMC1483603

[5] Roumeliotis S , Roumeliotis A , Dounousi E , Eleftheriadis T , Liakopoulos V . Dietary antioxidant supplements and uric acid in chronic kidney disease: a review. Nutrients. 2019;11(8):1911. 31443225 10.3390/nu11081911 PMC6723425

[6] Grassi G . Effects of serum uric acid on blood‐pressure lowering treatment. Curr Med Res Opin. 2017;33(sup3):15‐19. 28952389 10.1080/03007995.2017.1378520

[7] Kondo M , Imanishi M , Fukushima K , et al. Xanthine oxidase inhibition by febuxostat in macrophages suppresses angiotensin II‐induced aortic fibrosis. Am J Hypertens. 2019;32(3):249‐256. 30351343 10.1093/ajh/hpy157 PMC7110082

[8] Xie D , Zhao H , Lu J , et al. High uric acid induces liver fat accumulation via ROS/JNK/AP‐1 signaling. Am J Physiol Endocrinol Metab. 2021;320(6):E1032‐E1043. 33900847 10.1152/ajpendo.00518.2020

[9] Eliseev MS , Zheliabina OV , Nasonov EL . Uric acid, cognitive disorders, neurodegeneration. Ter Arkh. 2024;96(5):447‐452. 38829804 10.26442/00403660.2024.05.202698

[10] Kratzer JT , Lanaspa MA , Murphy MN , et al. Evolutionary history and metabolic insights of ancient mammalian uricases. Proc Natl Acad Sci USA. 2014;111(10):3763‐3768. 24550457 10.1073/pnas.1320393111 PMC3956161

[11] Li J , Li N , Wei J , et al. Genetically engineered mesenchymal stem cells with dopamine synthesis for Parkinson's disease in animal models. NPJ Parkinsons Dis. 2022;8(1):175. 36550118 10.1038/s41531-022-00440-6 PMC9780305

[12] Zhang S , Xu N , Fu L , et al. Integrated analysis of the complete sequence of a macaque genome. Nature. 2025;640(8059):714‐721. 40011769 10.1038/s41586-025-08596-w PMC12003069

[13] Tang DH , Wang CY , Huang X , et al. Inosine induces acute hyperuricaemia in rhesus monkey (*Macaca mulatta*) as a potential disease animal model. Pharm Biol. 2021;59(1):175‐182. 33715593 10.1080/13880209.2020.1871373 PMC7971274

[14] Johnson RJ , Sautin YY , Oliver WJ , et al. Lessons from comparative physiology: could uric acid represent a physiologic alarm signal gone awry in western society. J Comp Physiol B. 2009;179(1):67‐76. 18649082 10.1007/s00360-008-0291-7 PMC2684327

[15] Lottmann K , Chen X , Schädlich PK . Association between gout and all‐cause as well as cardiovascular mortality: a systematic review. Curr Rheumatol Rep. 2012;14(2):195‐203. 22350606 10.1007/s11926-011-0234-2 PMC3297741

[16] Kutzing MK , Firestein BL . Altered uric acid levels and disease states. J Pharmacol Exp Ther. 2008;324(1):1‐7. 17890445 10.1124/jpet.107.129031

[17] Wang L , Tan Z , Wang FY , Wu WP , Wu JC . Gout/hyperuricemia reduces the risk of Alzheimer's disease: a meta‐analysis based on latest evidence. Brain Behav. 2023;13(10):e3207. 37667521 10.1002/brb3.3207 PMC10570495

[18] Yang N , Xu L , Lin P , Cui J . Uric acid promotes neuronal differentiation of human placenta‐derived mesenchymal stem cells in a time‐ and concentration‐dependent manner. Neural Regen Res. 2012;7(10):756‐760. 25737698 10.3969/j.issn.1673-5374.2012.10.006 PMC4345657

[19] Prezioso D , Strazzullo P , Lotti T , et al. Dietary treatment of urinary risk factors for renal stone formation. A review of CLU working group. Arch Ital Urol Androl. 2015;87(2):105‐120. 26150027 10.4081/aiua.2015.2.105

[20] Kanbara A , Hakoda M , Seyama I . Urine alkalization facilitates uric acid excretion. Nutr J. 2010;9:45. 20955624 10.1186/1475-2891-9-45 PMC2976726

[21] Nakagawa T , Lanaspa MA , Johnson RJ . The effects of fruit consumption in patients with hyperuricaemia or gout. Rheumatology (Oxford). 2019;58(7):1133‐1141. 31004140 10.1093/rheumatology/kez128

[22] Pan J , Shi M , Li L , et al. Pterostilbene, a bioactive component of blueberries, alleviates renal fibrosis in a severe mouse model of hyperuricemic nephropathy. Biomed Pharmacother. 2019;109:1802‐1808. 30551434 10.1016/j.biopha.2018.11.022

[23] Hoque KM , Dixon EE , Lewis RM , et al. The ABCG2 Q141K hyperuricemia and gout associated variant illuminates the physiology of human urate excretion. Nat Commun. 2020;11(1):2767. 32488095 10.1038/s41467-020-16525-w PMC7265540

[24] Nagahara AH , Bernot T , Tuszynski MH . Age‐related cognitive deficits in rhesus monkeys mirror human deficits on an automated test battery. Neurobiol Aging. 2010;31(6):1020‐1031. 18760505 10.1016/j.neurobiolaging.2008.07.007 PMC2876823

[25] Zhang L , Yan J , Song S , et al. Detection of anxiety and depression‐like behavior and intra‐brain pathological markers in Parkinsonian cynomolgus monkeys. Exp Neurol. 2025;389:115242. 40194648 10.1016/j.expneurol.2025.115242

[26] Han R , Wang Q , Xiong X , et al. Deficiency of parkin causes neurodegeneration and accumulation of pathological α‐synuclein in monkey models. J Clin Invest. 2024;134(20). e179633. 39403921 10.1172/JCI179633 PMC11473153

[27] Mijailovic NR , Vesic K , Borovcanin MM . The influence of serum uric acid on the brain and cognitive dysfunction. Front Psychiatry. 2022;13:828476. 35530021 10.3389/fpsyt.2022.828476 PMC9072620

