# Supplementary figures and images for: Establishment of a novel cynomolgus monkey model of hyperuricemia
Source: Animal Model Exp Med. 2026 Jan 19;9(2):354–66. doi: 10.1002/ame2.70128 (PMC13042960; doi:10.1002/ame2.70128)

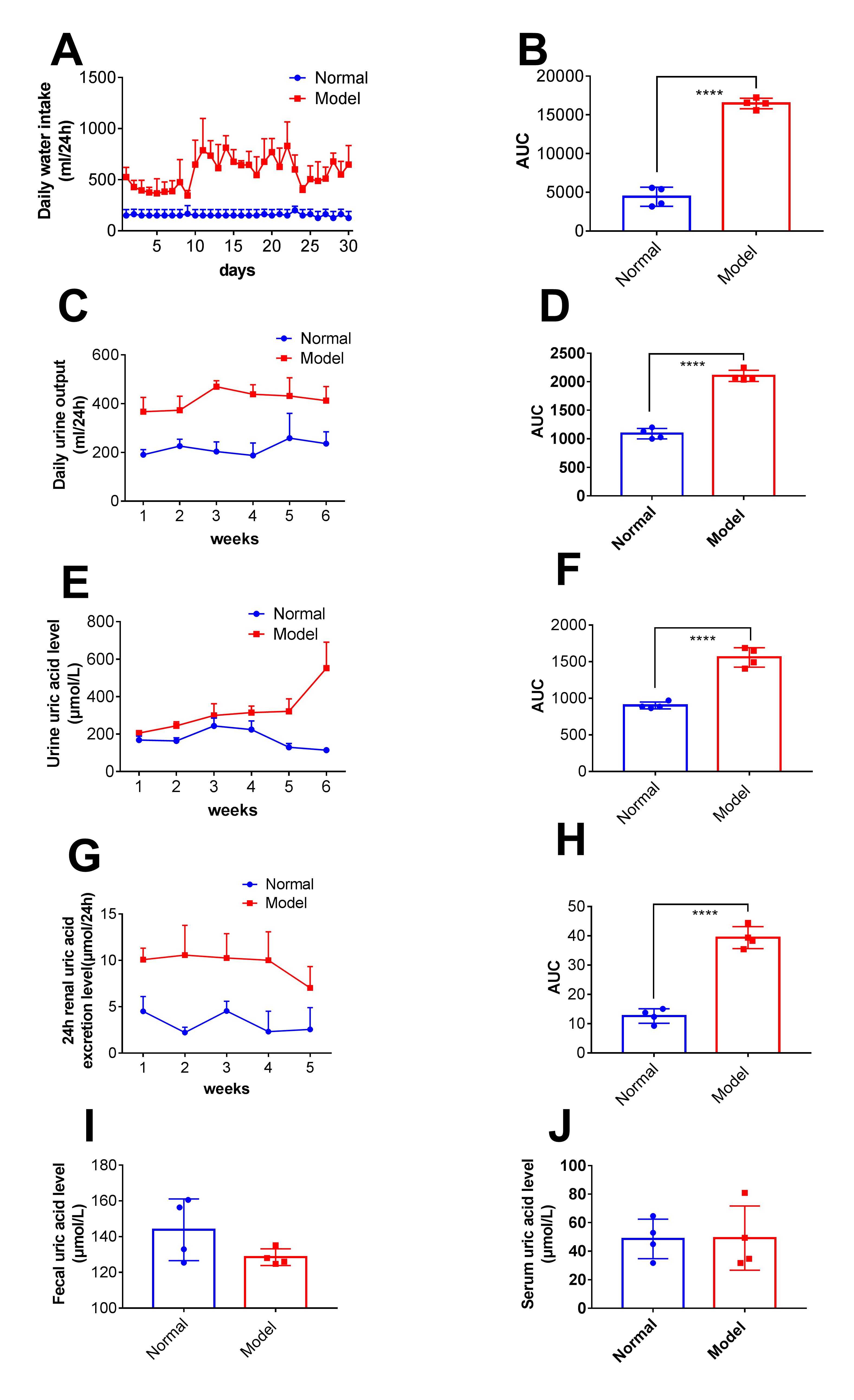

Supplement: Supplementary file 1 — Figure S1. [file AME2-9-354-s002.tif]

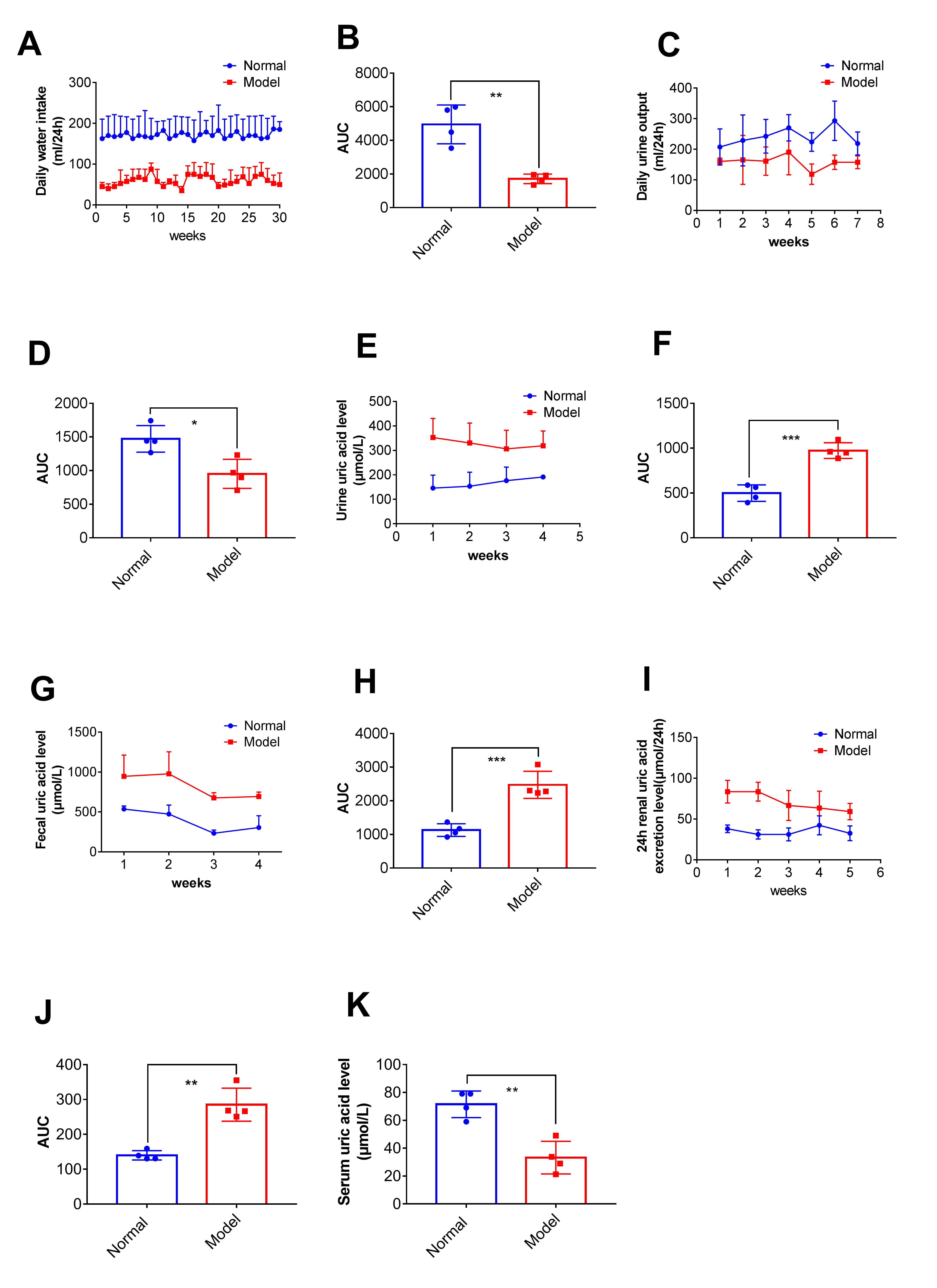

Supplement: Supplementary file 2 — Figure S2. [file AME2-9-354-s004.tif]

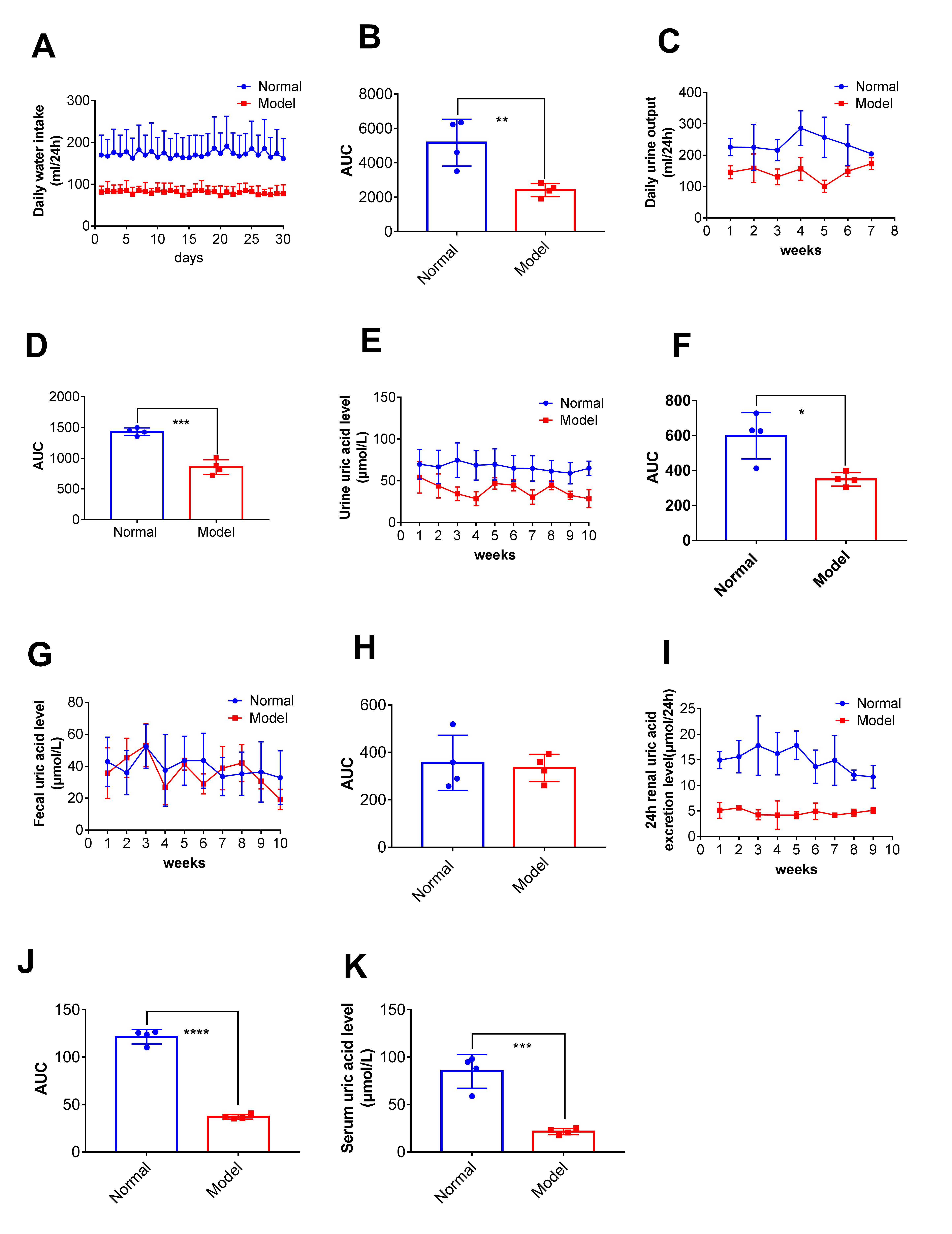

Supplement: Supplementary file 3 — Figure S3. [file AME2-9-354-s007.tif]

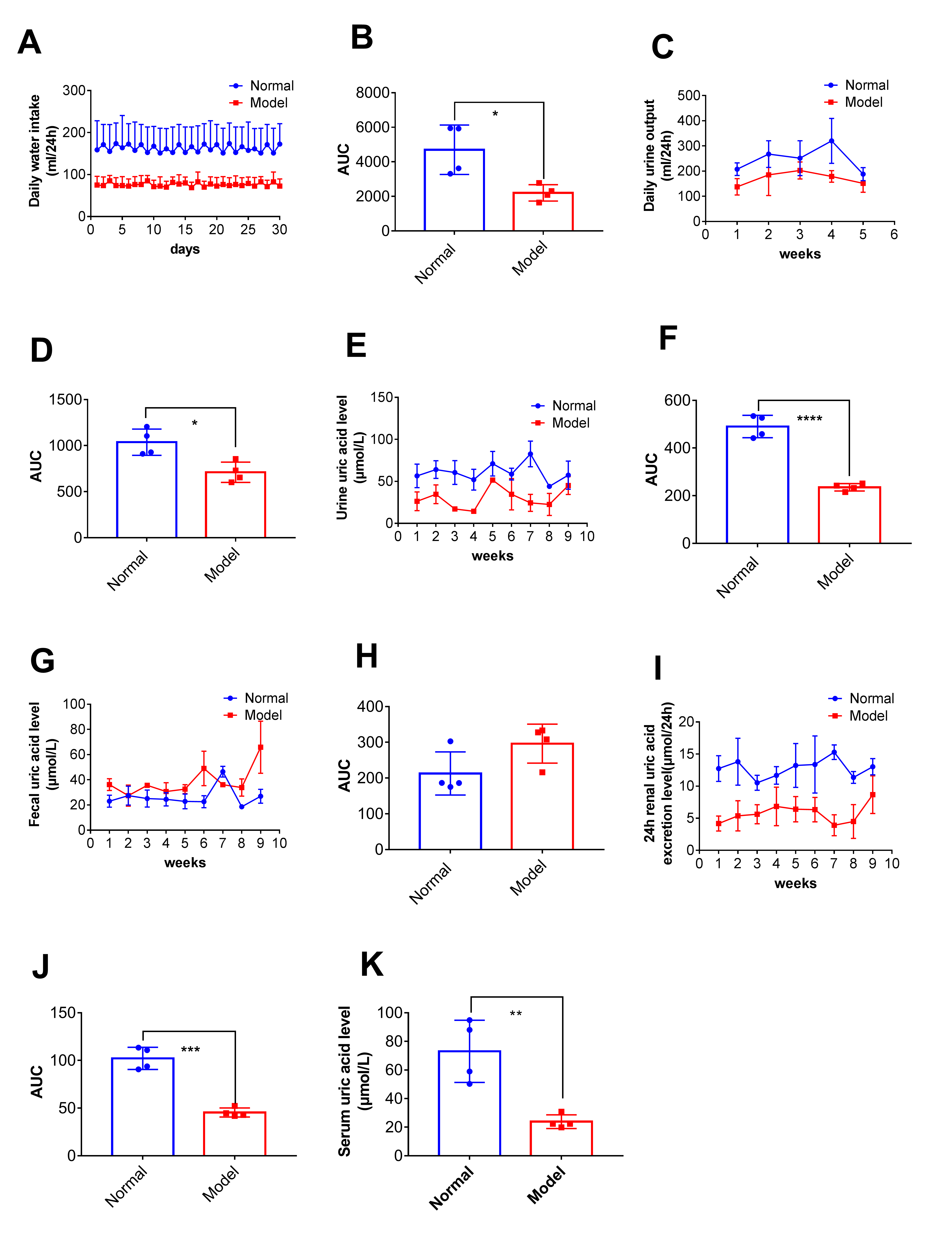

Supplement: Supplementary file 4 — Figure S4. [file AME2-9-354-s010.tif]

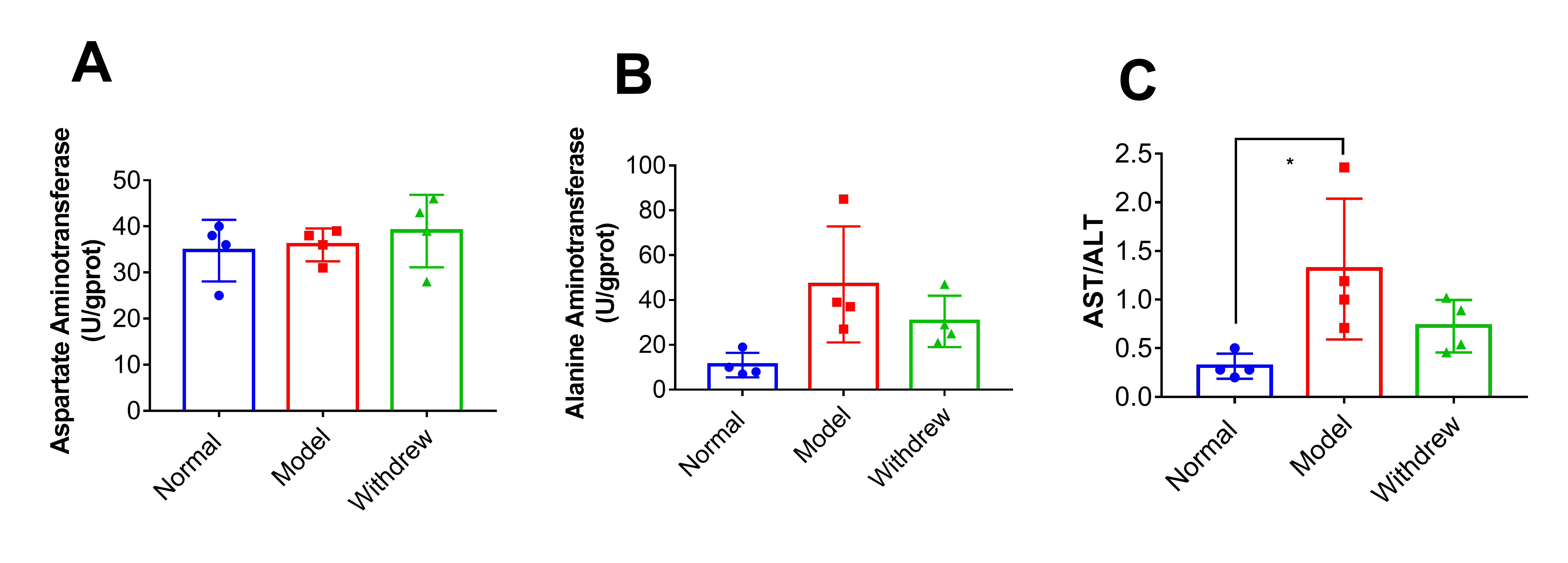

Supplement: Supplementary file 5 — Figure S5. [file AME2-9-354-s005.tif]
